# Comparative Analysis of P450 Signature Motifs EXXR and CXG in the Large and Diverse Kingdom of Fungi: Identification of Evolutionarily Conserved Amino Acid Patterns Characteristic of P450 Family

**DOI:** 10.1371/journal.pone.0095616

**Published:** 2014-04-17

**Authors:** Khajamohiddin Syed, Samson Sitheni Mashele

**Affiliations:** Department of Health Sciences, Faculty of Health and Environmental Sciences, Central University of Technology, Bloemfontein, Free State, South Africa; University of Missouri, United States of America

## Abstract

Cytochrome P450 monooxygenases (P450s) are heme-thiolate proteins distributed across the biological kingdoms. P450s are catalytically versatile and play key roles in organisms primary and secondary metabolism. Identification of P450s across the biological kingdoms depends largely on the identification of two P450 signature motifs, EXXR and CXG, in the protein sequence. Once a putative protein has been identified as P450, it will be assigned to a family and subfamily based on the criteria that P450s within a family share more than 40% homology and members of subfamilies share more than 55% homology. However, to date, no evidence has been presented that can distinguish members of a P450 family. Here, for the first time we report the identification of EXXR- and CXG-motifs-based amino acid patterns that are characteristic of the P450 family. Analysis of P450 signature motifs in the under-explored fungal P450s from four different phyla, ascomycota, basidiomycota, zygomycota and chytridiomycota, indicated that the EXXR motif is highly variable and the CXG motif is somewhat variable. The amino acids threonine and leucine are preferred as second and third amino acids in the EXXR motif and proline and glycine are preferred as second and third amino acids in the CXG motif in fungal P450s. Analysis of 67 P450 families from biological kingdoms such as plants, animals, bacteria and fungi showed conservation of a set of amino acid patterns characteristic of a particular P450 family in EXXR and CXG motifs. This suggests that during the divergence of P450 families from a common ancestor these amino acids patterns evolve and are retained in each P450 family as a signature of that family. The role of amino acid patterns characteristic of a P450 family in the structural and/or functional aspects of members of the P450 family is a topic for future research.

## Introduction

Fungi represent a large and diverse kingdom of lower eukaryotic organisms encompassing morphologically diverse yeasts, filamentous and dimorphic members. Members of this kingdom have adapted to diverse ecological niches, living as saprotrophs and obligate (or opportunistic) pathogens. Fungi play an important role in the cycling of elements in the biosphere and degradation of toxic environmental pollutants. Efforts by the Joint Genome Institute (JGI) of the US Department of Energy (US-DOE) as part of the fungal genomic program exploration of fungal diversity (http://genome.jgi.doe.gov/programs/fungi/index.jsf) [1] and the fungal genome initiative by the Broad Institute of Harvard and MIT [http://www.broadinstitute.org] resulted in sequencing of numerous under-explored fungal organisms; many fungal species genome sequencing projects are in progress.

Analysis of fungal genomes has revealed the presence of a surprisingly large number of cytochrome P450 monooxygenases (P450s), with some exceptions [2]–[4]. These enzymes are heme-thiolate proteins ubiquitously distributed across all biological kingdoms [5], with the highest numbers of genes in plants and lower numbers in fungi, animals, protists, bacteria, archaea and viruses (mimivirus) [4]. Despite their lower P450 gene counts, fungal genomes show the highest P450 diversity, with approximately 399 P450 families found across the 2784 annotated fungal P450s (as of 2011) and only 129 P450 families found across 4267 annotated plant P450s [4].

Apart from primary monooxygenation of substrates (incorporation of one oxygen atom into substrates), P450s perform a wide variety of reactions such as epoxidation, dealkylation, sulfoxydation, deamination, desulfuration, dehalogenation and nitro reduction in a stereo- and regio-selective manner [6], [7]. Reactivities essential to many pathways in the primary and secondary metabolism of fungal species include membrane ergosterol biosynthesis [8], [9], outer spore wall component biosynthesis [10], alkane and fatty acid degradation [11]–[13], fatty acid hydroxylation [14], mycotoxin synthesis (aflatoxins, trichothecenes and fumonisins) [15]–[17] and phytotoxin (gibberellins) synthesis [18]. Reactivities essential to the degradation of environmental pollutant species include modifications to carcinogenic and/or mutagenic polycyclic aromatic hydrocarbons and endocrine-disrupting chemicals [19]–[21].

Because of the large number of fungal genomes recently sequenced, several databases have been established to support the comprehensive and systematic analysis of P450s in fungal organisms [3], [22]–[24]. Identification of P450s in organisms is solely based on primary structure analysis of protein sequences, especially for the presence of two P450 signature motifs, FXXGXRXCXG (also known as CXG) in the heme-binding domain and the EXXR motif in the K-helix [25], [26]. The cysteine residue of the P450 signature CXG motif is invariantly conserved in all P450s, whereas the two glycines and one phenylalanine are generally, but not absolutely, conserved [25], [26]. The glutamic acid and arginine of EXXR motif are also conserved in P450s [25], [26] with few exceptions [27], [28].

The cysteine residue in the P450 signature motif CXG located in the β-bulge region (called Cys-pocket) serves as a fifth ligand to the heme iron. The first of the two glycine residues, which occurs four amino acids before the cysteine residue, allows for the formation of the β-hairpin turn; the second glycine residue, which occurs two amino acids after the cysteine residue, allows for a sharp turn in the backbone into the L-helix and for its positioning in proximity to the heme [29]. The EXXR motif is important for the stabilization of the meander loop and probably for the maintenance of the CYP tertiary structure [29], [30]. Site-directed mutagenesis of the invariant cysteine, glutamic acid or arginine in most CYPs resulted in the formation of completely inactive and misfolded P450 isoforms [31], [32], strongly suggesting that these invariant amino acid residues are critical for the folding and maintenance of the P450 structure.

Since their initial identification in P450s, structural analysis of these two signature motifs in the large numbers of P450 sequences now available has not been undertaken. Numerous studies describing amino acid residues important in substrate selectivity and regio selectivity have not highlighted any particular conservations in these signature motifs. Hence, we have begun systematic comparative analysis of EXXR and CXG motifs in the large collection of fungal P450s that is now available. In contrast to previous assumptions that amino acids in these motifs are extremely variable, our analyses on 12 fungal P450 family members showed predominant amino acids in these motifs. We found that particular amino acid patterns of EXXR and CXG motifs are characteristic of individual P450 families. Furthermore, analysis of 55 P450 families from different biological kingdoms including plants, animals, fungi and bacteria further strengthen the phenomenon of amino acid patterns at EXXR and CXG motifs characteristic of a P450 family. Results from this study have great implications for understanding the divergence of P450 families from a common ancestor and the role of these amino acid patterns, if any, in determining the substrate specificity or catalytic specificity of a P450 family.

## Materials and Methods

### Fungal Species

A detailed list of fungal species used in this study is listed in Table S1. In total 71 fungal species from four different fungal phyla, ascomycota (42 species), basidiomycota (26 species), zygomycota (two species) and chytridiomycota (one species) were used in this study. The phylum ascomycota comprises species from three sub-phyla: Saccharomycotina (22 species), Taphinomycotina (four species) and Pezizomycotina (16 species). All the species genome sequencing data are available for public use and proper procedures were followed for collection of data from the respective species genome data bases.

### Fungal Genome Mining for P450 Sequences

In total 4034 P450s from fungal phyla, ascomycota (1336 P450s), basidiomycota (2 859 P450s), zygomycota (102 P450s) and chytridiomycota (seven P450s) were used in this study. Ascomycota, zygomycota and chytridiomycota species P450s were obtained from the publicly available Cytochrome P450 Homepage (http://drnelson.uthsc.edu/P450seqs.dbs.html) [3], except for the two thermophilic biomass-degrading species, *Thielavia terrestris* and *Myceliophthora thermophila;* P450s were obtained from an author’s (Dr Syed) recent work [33].

Phylum basidiomycota species P450s for *Phanerochaete chrysosporium*, *Ganoderma* sp., *Bjerkandera adusta* and *Phlebia brevispora* were obtained from an author’s (Dr Syed) own work that has been published and is available for public use [34], [35]. P450s for *Postia placenta* were obtained from Ide et al. [36]. P450s for *Agaricus bisporus* and *Serpula lacrymans* were obtained from the fungal cytochrome P450 database (FCPD; http://p450.riceblast.snu.ac.kr/index.php?a=view) [24]. P450s for *Cryptococcus gattii, Malassezia globosa, Puccinia graminis* and *Sporobolomyces roseus* were obtained from the publicly available Cytochrome P450 Homepage [3]. P450s for the softwood-degrading basidiomycete *Phanerochaete carnosa* were retrieved from published literature [37] to which an author (Dr Syed) contributed the P450 section as part of the P450 annotation team. The medicinal mushroom *Ganoderma lucidum* [38] P450s were kindly provided by Dr Nelson, University of Tennessee, USA. In the remaining 12 basidiomycete species, i.e. *Auricularia delicate*, *Coniophora puteana*, *Dacryopinax* sp., *Dichimotus squalene*, *Fomitiporia mediterranea*, *Fomitopsis pinicola*, *Gloeophyllum trabeum, Punctularia strigosozonata*, *Stereum hirsutum*, *Trametes versicolor*, *Tremella mesenterica Fries* and *Wolfiporia cocos*, P450s were identified following the standard procedure as described by Dr Syed in his recent publications [33], [35] and his contribution (as part of the P450 annotation team) in fungal genome sequencing articles published in authoritative scientific journals [37], [39], [40] with a major modification of the method. The new strategy followed for identification and annotation of P450s is: protein sequences from each of the fungal species were downloaded from the respective species website at the JGI of the US-DOE (http://genome.jgi.doe.gov/programs/fungi/index.jsf) [1]. The downloaded protein sequences were submitted to the National Center for Biotechnology and Information Conserved Domain Database for the functional annotation of proteins: NCBI *Batch* Web CD-search tool (http://www.ncbi.nlm.nih.gov/Structure/bwrpsb/bwrpsb.cgi) [41]–[43]. This program identifies and segregates putative proteins into different protein families based on protein families characteristic conserved domains. The output data were compared in tabular form and the putative protein sequences grouped under the P450 superfamily were selected for further analysis (Table S2). The selected proteins were analyzed for the presence of the P450 family signature motifs, namely the EXXR and CXG. The proteins that showed both motifs were considered as authentic P450s and used in this study.

### Selection of P450s

All P450 sequences used in this study followed Dr Nelson’s nomenclature [44]–[46]. The recently revised FCPD provided P450 nomenclature equivalent to Dr Nelson’s nomenclature for basidiomycete species, *A. bisporus* and *S. lacrymans*. Therefore, the P450s downloaded from FCPD for both basidiomycete species could be used directly in this study. For the remaining 12 basidiomycete species (listed above), the identified P450s were subjected to blast analysis against all named fungal species at the Cytochrome P450 webpage [3]. For each P450 the closest homolog was identified and based on the homology percentage, family and subfamily names were assigned. For assigning the family and subfamily names, the standard rule set by the International P450 Nomenclature Committee was followed, i.e. P450s within a family share more than 40% amino acid identity and members of subfamilies share more than 55% amino acid identity [44]–[46]. As a strict rule, pseudo-P450s and alleles from each of the species were excluded from this analysis.

### P450 Signature Motifs EXXR and CXG Analysis in Fungal P450s

In order to analyze the P450 signature domains in the selected fungal P450s, we performed ClustalW analysis using Molecular Evolutionary Genetics Analysis (MEGA 5.2.2) software [47]. The advantage of using MEGA-based ClustalW is that this program combines both pairwise alignment and multiple alignment as part of ClustalW.

The ClustalW-aligned P450 sequences were analyzed for amino acid patterns in the P450 signature motifs EXXR and CXG. The amino acid residues in P450 signatures were selected from the ClustalW program from MEGA and computed into tabular form. After sorting to ascending order, manual analyses were performed to check the type of amino acids and their count in P450 signature motifs. The proportions of types of amino acids were calculated and presented in both pie charts and tables.

Some P450s showed variations of the EXXR and CXG motifs. The same phenomenon was also reported in the literature [5], [48], [49]. Authors have suggested that these P450s may be misaligned or that the P450s are missing the invariant residues at the EXXR and CXG motif. This is unlike the *Streptomyces* species P450s that did not contain the conserved EXXR domain but rather EVLW and EQILW [27], [28], which had been proved to be functional. Owing to the lack of functional data with regard to the fungal P450s, which lack the EXXR and CXG motifs signature amino acids; we excluded these P450s from this analysis.

### P450 Family-level-based P450 Signature Motifs Analysis

In order to analyze the P450 family level organization of the P450 signature motif, we selected 67 P450 families from all biological kingdoms (animals, plants, bacteria and fungi). Member P450s belonging to 55 P450 families of animals, plants and bacteria were downloaded from CYPED (http://www.cyped.uni-stuttgart.de/cgi-bin/CYPED5/index.pl) [26]. The downloaded sequences were subjected to P450 signature analysis as described in the above section. During ClustalW analysis some of the P450 sequences (35 P450s) that are short in length (<300 amino acids) were manually deleted and the rest of the sequences were used for P450 signature motif analysis.

Members of the fungal P450 families were poorly represented on the CYPED website and many fungal P450 families were not represented, especially the P450 families that are highly populated in basidiomycete fungi capable of degrading wood components [50]. Hence, in this study, we followed two ways to collect members of fungal P450 families. First, member P450s belonging to fungal P450s were retrieved from the published literature (see section fungal genome mining for P450 sequences), where P450s were annotated as per Dr Nelson’s nomenclature [44]–[46]. The P450 sequences from 12 basidiomycete species (for list of fungal species see section “Fungal genome mining for P450 sequences”) were subjected to family and subfamily classification in the same way as described in the section “Selection of P450s” and member P450s belonging to different fungal P450 families were selected for analysis. Fungal P450 families such as such as CYP52, CYP61, CYP63, CYP512, CYP5035, CYP5037, CYP5136, CYP5139, CYP5141, CYP5144, CYP5150 and CYP5152 were included in this study. The criteria for selection of these P450 families are based on the facts that (i) recent study in our laboratory showed enrichment of some of these P450 families in fungal species [50] and (ii) there is evidence of conservation of certain P450 families across the fungal species. Analysis of fungal P450 families and their member P450s in fungal species and the literature consulted for retrieving the member P450s were presented in Table 1 and Table S1. The P450 families selected for analysis of P450 signature motifs followed a strict rule that the number of member P450s should be close to 100 P450s, with some exceptions for fungal P450 families (Table 1). The reason for a minimum of 100 members of a P450 family being included in the analysis is that they represent the diversity and hence true amino acid pattern in the P450 signature motifs. Analysis of P450 signature motifs in members of P450 families was performed as described in the above section.

**Table 1:** Comparative analysis of member P450s in selected P450 families across 21 basidiomycete species.

	CYP63	CYP512	CYP5035	CYP5037	CYP5136	CYP5141	CYP5144	CYP5150	CYP5139	CYP5152	References
*Phanerochaete chrysosporium*	7	14	13	5	5	8	35	7	1	2	34, 56
*Postia placenta*	5	15	3	14	0	4	4	32	8	2	36
*Phanerochaete carnosa*	9	14	14	5	5	6	48	10	8	4	37
*Bjerkandera adusta*	5	16	5	5	6	8	54	15	2	3	35
*Ceriporiopsis subvermispora*	13	10	9	15	12	2	48	8	14	6	40
*Ganoderma* sp.	5	18	13	5	9	2	4	32	5	1	35
*Ganoderma lucidum*	13	21	15	6	7	2	3	36	7	1	38
*Phlebia brevispora*	6	5	10	9	2	5	8	18	4	2	35
*Agaricus bisporus*	6	12	0	3	0	7	14	12	4	0	24 and this work
*Serpula lacrymans*	7	11	0	0	5	5	25	2	6	11	24 and this work
*Stereum hirsutuma*	10	10	3	30	4	2	38	24	13	1	This work
*Trametes versicolora*	5	8	6	10	3	3	42	39	12	2	This work
*Wolfiporia cocosa*	8	19	2	37	2	3	9	28	10	1	This work
*Auricularia delicatea*	7	14	4	27	0	15	55	6	20	0	This work
*Coniophora puteanaa*	7	6	1	6	1	1	57	none	22	23	This work
*Dacryopinax* sp.	0	6	1	10	1	2	13	0	12	2	This work
*Dichimotus squalenea*	6	14	12	7	5	3	41	21	8	1	This work
*Fomitiporia mediterraneaa*	3	13	9	14	2	4	11	9	5	1	This work
*Fomitopsis pinicolaa*	5	14	3	30	3	2	7	28	11	2	This work
*Gloeophyllum trabeuma*	3	8	1	17	0	0	14	6	10	1	This work
*Punctularia strigosozonataa*	5	4	4	12	0	2	21	15	13	3	This work

Member P450s belonging to different P450 families in basidiomycetes were collected using three methods. (i) P450s belonging to different families were retrieved from published data bases (indicated with reference number in the table). (ii) P450 members belonging to different families were identified by being annotated and assigned to families and subfamilies as described in the “Materials and methods” section (indicated by “this work” in the table). (iii) Some basidiomycete P450s were refined from the published literature (indicated with reference and “this work” in the table). Members of P450 families that are absent in different basidiomycete species were indicated with “0″ in the table.

### Generation of P450 Family Characteristics Sequence Logos

Sequence logos are a graphical representation of an amino acid/nucleic acid multiple sequence alignment that displays patterns in sequence conservation [51], [52]. A sequence logo consists of stacks of symbols. Each stack represents a single position in the sequence. The height of the stack indicates the sequence conservation at that position, while the height of symbols within the stack indicates the relative frequency of each amino/nucleic acid at that position. Protein logos unravel the patterns of amino acid conservation that are often of structural or functional importance [53], [54].

In the present study we used WebLogo: A sequence logo generator programme (http://weblogo.berkeley.edu/logo.cgi) [51], [55] to create protein logos at the EXXR and CXG motifs for each of the P450s family. After ClustalW alignment of member P450s in each P450 family, the EXXR and CXG (FXXGXRXCXG) region amino acids were selected and pasted in the WebLogo program. As a selection parameter, image format was selected as PDF and 32 symbols per line were selected. The generated EXXR and CXG logos were used for the analysis.

## Results and Discussion

For the last five decades research has been focused on cytochrome P450 monooxygenases to harness their potential for pharmacological, biotechnological and environmental applications. Recent studies on fungal organisms revealed the presence of a large number of P450 contingents in their genomes, with some exceptions [4], [24], [39]. Subsequent functional studies suggested that fungal P450s are catalytically diverse [19], [36], [56] and perform extraordinary oxidation activity compared to P450s across the biological kingdoms [21]. The latest study conducted in our laboratory revealed the presence of a large number of thermostable P450s with biotechnological potential in fungi [33]. Apart from these important findings, recent results from our laboratory identified P450 families enriched in fungal species, especially in wood-degrading basidiomycetes that are predicted to be involved in fungal adaptation and colonization to diverse ecological niches [50].

In view of the large and diverse nature of the fungal kingdom, presence of a large number of P450s in fungal species and under-representation of fungal P450s in terms of structure-activity studies, in this study we selected fungal P450s to assess the nature of the conserved P450 signature motifs EXXR and CXG.

### Comparative Analysis of P450 Signature Motifs EXXR and CXG in Fungal P450s

A recent study by the Jürgen Pleiss group [57] identified a large number of conserved amino acid residues in P450s. This study involved comparing the P450s of two different classes that are classified based on electron donors (class I P450s accepting electrons from ferredoxin and class II P450s accepting electrons from CPR-type reductase). However, in this study, conservation of amino acids or predominance of a certain amino acid patterns within the P450 signature motifs, such as EXXR or CXG and/or family level conservation of amino acid positions in these motifs, is not reported, except for one new observation of the predominance of glycine after phenylalanine amino acid at the CXG motif in both classes of P450s [57]. Furthermore, fungal P450 families were under-represented in terms of the structure-activity relationship. Hence, in the present study we focused on the analysis of amino acids within the EXXR and CXG (between C and G amino acids) motifs in the newly identified and publicly available fungal P450s and their families.

### Analysis of EXXR Motif

**Figure 1:**
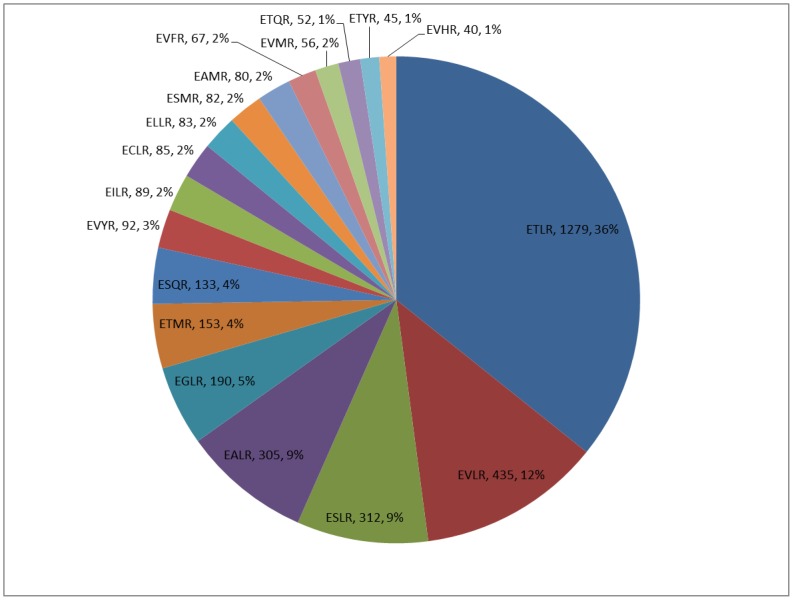
Comparative analysis of amino acid patterns in P450 signature motif EXXR in fungal P450s. A total of 4304 P450s from the fungal phyla ascomycota (1336 P450s), basidiomycota (2859 P450s), zygomycota (102 P450s) and chytridiomycota (7 P450s) were used for the analysis. A total of 128 amino acid patterns for the EXXR motif were identified. The predominant amino acid patterns (>2%) are shown in the pie charts. Amino acid patterns are shown in the figure along with the number of amino acids representing the pattern and overall percentage of that pattern. A detailed list of amino acid patterns occurring at P450 signature motifs is shown in Table S3.

Analysis of 4304 P450s from four fungal phyla, ascomycota, basidiomycota, zygomycota and chytridiomycota, revealed 128 amino acid patterns for the EXXR signature motif (Fig. 1 and Table S3). Among the 128 amino acid patterns observed for the EXXR motif, only a few patterns are predominant (Fig. 1), including ETLR found dominantly in 1279 sequences (36% of all analyzed sequences), EVLR found in 435 sequences (12%), ESLR and EALR found in 312 and 305 sequences (9% each). Comparison of the EXXR motif between the P450s of individual fungal phyla ascomycota and basidiomycota revealed predominance of the same pattern of amino acids as observed for all fungal P450s (Table S4). While ETLR is found to be the most predominant pattern across the fungal phyla, differences were observed between the fungal phyla in the rest of the predominant amino acid patterns. Amino acid pattern ESLR is the second most predominant pattern in basidiomycota, whereas it is the fourth predominant pattern in ascomycota (Table S4). The amino acid pattern EVLR was the second most predominant in ascomycota, whereas it is the fourth predominant pattern in basidiomycota (Table S4). It is noteworthy that 24 amino acid patterns are represented by a single P450 and 17 amino acid patterns are represented twice in the collection of 4304 sequences, suggesting that these patterns are rare exceptions (Table S3).

Among 128 amino acid patterns found for the EXXR motif, glutamic acid and arginine were found to be the first and last positions. Identification of glutamic acid and arginine at the first and last positions in this motif is well reported in the literature [25], [26]. Results from this study using a large number of fungal P450s further strengthen the conclusion that glutamic acid and arginine are conserved residues in the EXXR motif in P450s across the biological kingdoms. Analysis of fungal P450s revealed the occurrence of 15 amino acids (A/C/E/F/G/I/L/M/N/P/Q/S/T/V/W) (Table 2) at the second position in this motif. The remaining five amino acids (D/H/K/R/Y) never appeared in fungal P450s analyzed in this study at the second position in this motif. All amino acids appeared at the third position in this motif (Table 2). Overall, our analysis suggests that threonine (amino acid with polar neutral side chain) and leucine (amino acid with hydrophobic side chain-aliphatic) are preferable as second and third amino acid residues in the EXXR motif (Table 2). Comparison of the number of amino acid patterns between the fungal phyla revealed the presence of a higher number of amino acid patterns in basidiomycota (Table S4). It is noteworthy that despite the high diversity of P450s found in ascomycota [4], a low number of amino acid patterns were found for this motif.

**Table 2:** Analysis of P450 signature motifs EXXR and CXG in fungal P450s.

EXXR						CXG					
Second position			Third position			Second position			Third position		
Appear	No. ofP450s	percentage	Appear	No. ofP450s	percentage	Appear	No. ofP450s	percentage	Appear	No. ofP450s	percentage
T	1658	38.52	L	2811	65.31	P	1678	38.99	G	4199	97.56
V	767	17.82	M	416	9.67	I	1238	28.76	A	97	2.25
S	628	14.59	Q	239	5.55	L	719	16.71	D	2	0.05
A	490	11.38	Y	187	4.34	V	325	7.55	K	2	0.05
G	244	5.67	F	181	4.21	A	131	3.04	E	1	0.02
L	161	3.74	I	128	2.97	S	57	1.32	P	1	0.02
C	153	3.55	H	94	2.18	T	33	0.77	Q	1	0.02
I	134	3.11	V	60	1.39	Q	32	0.74	S	1	0.02
N	35	0.81	S	42	0.98	M	28	0.65	R	0	0
M	21	0.49	A	36	0.84	G	21	0.49	I	0	0
F	6	0.14	E	25	0.58	K	11	0.26	T	0	0
E	4	0.09	T	24	0.56	F	9	0.21	V	0	0
P	1	0.02	G	22	0.51	E	6	0.14	C	0	0
Q	1	0.02	C	15	0.35	W	4	0.09	F	0	0
W	1	0.02	N	9	0.21	Y	4	0.09	H	0	0
D	0	0	W	6	0.14	H	3	0.07	L	0	0
Y	0	0	R	5	0.12	R	3	0.07	M	0	0
H	0	0	P	2	0.05	C	1	0.02	N	0	0
R	0	0	D	1	0.02	N	1	0.02	W	0	0
K	0	0	K	1	0.02	D	0	0	Y	0	0

Comparative analysis of 4304 P450s from fungal phyla ascomycota, basidiomycota, zygomycota and chytridiomycota revealed amino acids that are part of the fungal P450 signature motifs. Amino acids appearing in the P450 signature motifs were represented from top to bottom with their frequencies at each position.

### Analysis of CXG Motif

**Figure 2:**
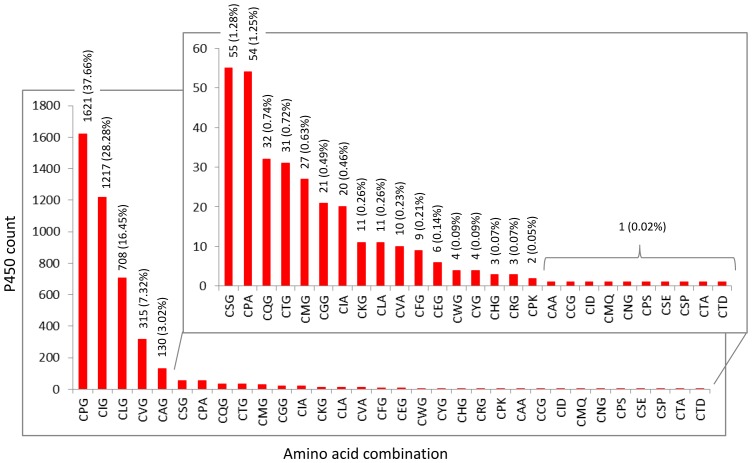
Comparative analysis of amino acid patterns at the P450 signature motif CXG in fungal P450s. In total 4304 P450s from the fungal phyla ascomycota (1336 P450s), basidiomycota (2859 P450s), zygomycota (102 P450s) and chytridiomycota (seven P450s) were used for the analysis. As shown in the figures, 32 amino acid patterns were identified in fungal P450 for the CXG motif. The numbers on the bars represent the number of amino acids representing the pattern. The overall percentage of that pattern is shown in parenthesis.

Unlike 128 amino acid patterns observed for the EXXR motif, only 32 patterns were found for the CXG motif (Fig. 2 and Table S3). Consistent with the earlier studies [25], [26], our analysis of fungal P450s also found cysteine to be the only invariant amino acid in this motif. While 19 amino acids occur as the second amino acid in this motif aspartic acid never present as second amino acid, glycine and seven other amino acids (A/D/E/K/P/Q/S) occur as the third amino acid in this motif (Table 2). The remaining 12 amino acids (Table 2) are never present as third amino acid in this motif. The variant nature of the second amino acid in this motif is well-documented in the literature [25], [26]. However, the predominance of particular amino acids as second amino acid in this motif is not reported. Furthermore, the occurrence of alternative amino acids at the third position in this motif has not been reported. Hence, this study constitutes the first report on the identification of predominant amino acids as second amino acid and identification of alternative amino acids as third amino acid in this motif in fungal P450s. Analysis of amino acids at the CXG motif revealed that proline is the predominant amino acid (39%) at the second position, followed by isoleucine (29%) and leucine (17%); glycine is the predominant amino acid (97%) as third amino acid in the CXG motif (Table 2). Alanine is represented by 97 amino acids (2%); aspartic acid and lysine by two P450s each and the rest of the amino acids by a single P450 (Table 2). Analysis of P450s representing the other amino acids in place of glycine at the CXG motif suggested that these P450s are true P450s since they possess the EXXR motif and contain cysteine as prime amino acid at the CXG motif.

Among the 32 amino acid patterns observed in this motif, three patterns were predominant across the fungal P450s (Fig. 2). CPG is the most predominant pattern found in 1621 sequences (38%), followed by CIG found in 1217 sequences (28%) and CLG found in 708 sequences (16%). This suggests that proline is highly preferred as the second amino acid in this motif in fungal P450s. Ten unique patterns represented by single P450 were observed for this motif (Table S3). It is interesting that a clear difference in preference of amino acid patterns was observed between two fungal phyla (Table S4). Ascomycota P450s showed CIG and CLG as predominant amino acid pattern (30% each), followed by CPG (19%). However, basidiomycota P450s showed CPG as predominant amino acid pattern (46%), followed by CIG (27%) and CLG (10%) (Table S4). Further differences were observed between the two phyla on the appearance of a number of amino acid patterns (Table S4). As shown in Table S4, 19 amino acid patterns were common to both the fungal phyla ascomycota and basidiomycota. Ten and four patterns were unique to the ascomycota and basidiomycota, respectively. Overall, compared to basidiomycota, ascomycota showed rich diversity in terms of the number of amino acid patterns at the CXG motif. The presence of a large number of P450 families in ascomycota, as described in previous studies [4], might contribute to the rich diversity at the CXG motif. A small number of P450 families and the occurrence of higher levels of P450 gene duplications in basidiomycota, as recently reported from our laboratory [50], contributed to the low diversity of amino acid patterns at CXG motifs.

Considering the above marked difference between the fungal phyla ascomycota and basidiomycota and within ascomycota, both in preference of amino acids at second position and number of patterns at P450 signature motifs (Table S4), we checked the range of P450 families contributing to the predominant amino acid patterns in each of these phyla further.

### Fungal P450 Families show Characteristic Amino Acid Pattern at P450 Signature Motifs

From the above study it is clearly evident that particular amino acid patterns are predominant at P450 signature motifs, especially at the CXG motif, among different fungal phyla (Table S4). For example, P450s of Pezizomycotina species showed CIG as the predominant amino acid pattern (31%) followed by CLG (23%) and CPG (22%), whereas P450s from Saccharomycotina and Taphinomycotina showed CLG as the most predominant amino acid pattern (75%), followed by CIG (17%) (Table S4). P450s from basidiomycota species showed CPG (46%) as the predominant amino acid pattern at the CXG motif (Table S4). Analysis of the P450 families present in 26 species from Saccharomycotina (23 species) and Taphinomycotina (three species) showed only 11 P450 families in these subphyla, whereas 16 species of Pezizomycotina showed 298 P450 families in this subphylum. The lower P450 diversity observed for Saccharomycotina and Taphinomycotina is due to the reduced number of P450s in their genomes and the small number of P450 families containing more than one P450 (e.g., CYP51, CYP52, CYP56, CYP61 and CYP501). This suggests that the CLG pattern dominates in these particular P450 families. As Pezizomycotina species contain a significantly higher number of P450 families (298 P450 families) in their genomes compared to basidiomycota (105 P450 families), it is understandable that multiple amino acid patterns occur within their many P450 families.

**Figure 3:**
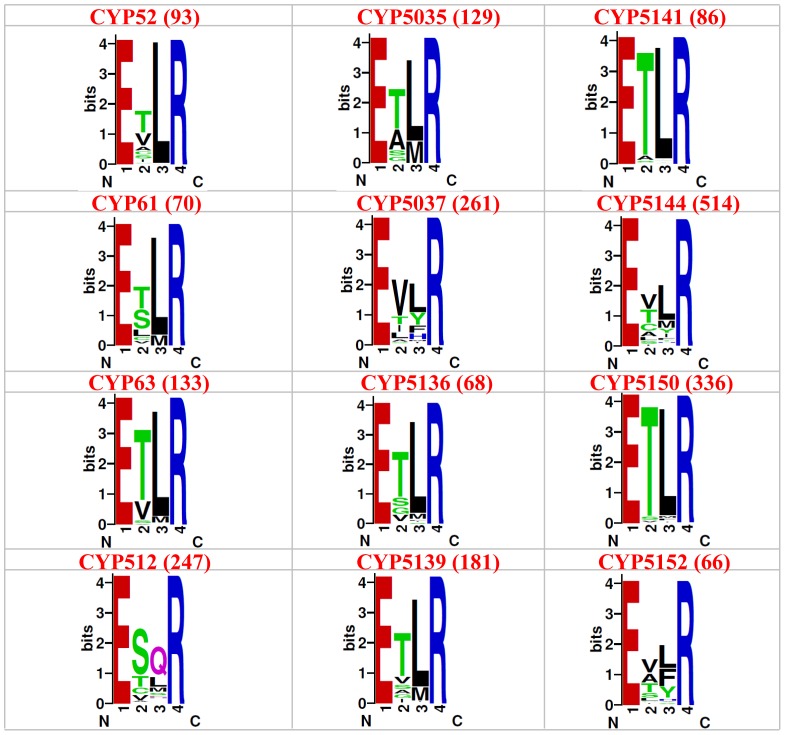
Analysis of amino acid patterns at EXXR motif in 12 fungal P450 families. A sequence logo for the EXXR motif using the amino acids from 12 fungal P450 families was constructed, as described in the “Materials and methods” section. The number of P450s used for the construction of the sequence logo is shown in the parenthesis right next to the name of the P450 family.

**Figure 4:**
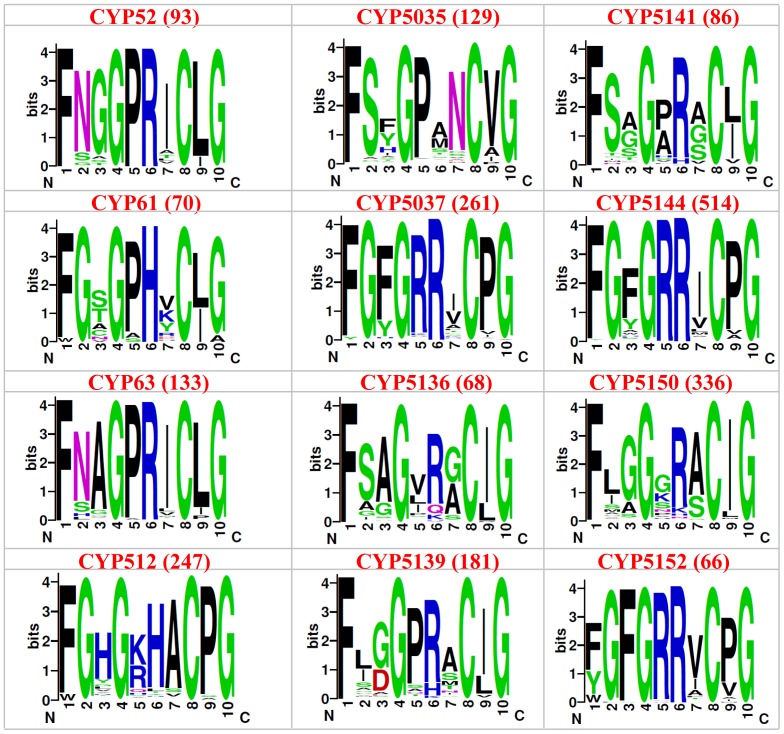
Analysis of amino acid patterns at CXG motif in 12 fungal P450 families. Sequence logo for the CXG motif (FXXGXRXCXG) using the amino acids from 12 fungal P450 families were constructed as described in the “Materials and methods” section. The number of P450s used for construction of the sequence logo is shown in the parenthesis right next to the name of the P450 family.

In order to unravel the particular amino acid patterns prevailing in a particular P450 family (characteristic of that P450 family), if any, we constructed sequence logos for the EXXR and CXG motif for 12 fungal P450 families (Figures 3 and 4). The P450 families analyzed in this study include the P450 families that are conserved across the fungal species, such as CYP61, enriched in both ascomycota (CYP52) and in basidiomycota (CYP63, CYP512, CYP5035, CYP5037, CYP5136, CYP5139, CYP5141, CYP5144, CYP5150 and CYP5152) [50]. The sequence logo for CXG motifs covered the entire motif, i.e. FXXGXRXCXG. Sequence logos are very helpful to identify a particular amino acid dominance/conservation at a particular position instantly [51], [52]. As shown in Figures 3 and 4, certain amino acid patterns look conserved or predominant in the EXXR and CXG motifs across the P450 families. A detailed analysis of the percentage conservation of the amino acid at each position in the motifs is presented in Table 3.

**Table 3:** Comparative quantitative analysis of amino acid patterns at EXXR and CXG (FXXGXRXCXG) motifs in 12 fungal P450 families.

P450 family	Number ofMember P450s	E-X-X-R	F-X-X-G-X-R-X-C-X-G
CYP52	93	E-T(42)/V(24)-L(99)-R	F-N(85)-G(83)-G-P-R-I(79)-C-L(90)-G
CYP61	70	E-T(26)/S(24)-L(90)-R	F(96)-G-S(34)/T(29)-G-P(91)-V(53)-C-L(61)-G(93)
CYP63	133	E-T(74)-L(92)-R	F-N(78)-A(89)-G-P(98)-R(99)-I(87)-C-L(86)-G
CYP512	247	E-S(62)-Q(53)-R	F(94)-G(99)-H(69)-G(99)-K(47)-H(87)-A(88)-C-P(95)-G
CYP5035	129	E-T(54)-L(78)-R	F(98)-S(93)-F(31)/Y(29)-G(99)-P(94)-A(39)/M(23)-N(85)-V(81)-G(99)
CYP5037	261	E-V(56)-L(48)/Y(20)-R	F(96)-G(99)-F(79)-G(99)-R(99.6)-I(38)/V(28)-C-P(90)-G(98)
CYP5136	68	E-T(63)-L(61)-R	F-S(74)-A(76)-G-V(44)/L(19)-R(79)-G(49)/A(41)-C-I(79)-G
CYP5139	181	E-T(62)-L(84)-R	F-L(40)/I(25)-G(62)-G-P(83)-R(83)-A(49)/S(16)-C-I(73)-G
CYP5141	86	E-T(92)-L(95)-R	F-S(89)-A(35)/G(33)-G-P(47)/A(37)-R(93)-A(36)/G(30)-C-L(47)/I(45)-G
CYP5144	514	E-V(29)/T(26)-L(58)-R(99.6)	F(97)-G(99)-F(68)-G(99)-R(99)-R(99.8)-I(65)-C-P(88)-G(99.8)
CYP5150	336	E-T(94)-L(97)-R(99)	F(99)-L(49)/I(18)-G(77)-G(99)-G(40)/K(19)-R(85)-A(68)-C-I(90)-G
CYP5152	66	E-V(30)/A(14)-L(38)/F(30)-R	F(59)-G-F-G-R(98)-R-V(66)-C-P(77)-G

The percentage predominance of amino acids at particular positions is calculated considering the total number of amino acids as 100%. Amino acids or patterns of amino acids contributing more than 50% at the specific position are shown in the table. Amino acids conserved (100%) at the specific position(s) are represented by their symbol. The numerical values in the table are percentage values.

Comparison of the EXXR motif across the fungal P450 families suggested high conservation of particular amino acids, such as threonine (T) and leucine (L) at the second and third positions, with some exceptions (Fig. 3 and Table 3). The CYP52 P450 family contained serine (S) as the predominant amino acid and valine (V) is the predominant amino acid in CYP5037 as second amino acid. Furthermore, the CYP512 P450 family showed glutamine (Q) and CYP5152 showed leucine (L) and phenylalanine (F) as predominant third amino acid (Table 3) at this motif. Overall, compared to other fungal P450 families, CYP512 and CYP5152 P450 families showed amino acid patterns characteristic of the P450 family at this motif (Fig. 3 and Table 3).

Analysis of the CXG motif across the 12 fungal P450 families (Fig. 4 and Table 3) revealed conservation of the amino acid patterns characteristic of a P450 family, indicating their common ancestral origin. In comparison to the consensus sequence FXXGXRXCXG [25], [26], at this motif differences were observed for each P450 family in terms of conservation of specific amino acids reflecting the nature of P450 family evolution (divergence evolution). Analysis of the second amino acid in this motif, which is designated as “X (any amino acid)”, suggested that glycine is conserved or predominant in P450 families, CYP61, CYP512, CYP5037, CYP5144, and CYP5122; aspargine (N) is predominant in P450 families CYP52 and CYP63; serine is predominant in P450 families, CYP5035, CYP5136 and CYP5144 (Fig. 4 and Table 3). A recent study by the Jüergen Pleiss group [57] also showed glycine as the predominant amino acid at this position. However, this study did not report other amino acids or family level conservation of specific amino acids at this position [57]. The sixth position amino acid in CYP61 and CYP512 is histidine (H) rather than arginine (Fig. 4 and Table 3). The same goes for the rest of the positions that are designated as “X” in the consensus sequence. An interesting observation among P450 families is the conservation or predominance of a particular amino acid between cysteine (C) and glycine (G) among P450 families (Fig. 4 and Table 3). Leucine (L) is the predominant amino acid in P450 families CYP52, CYP61, CYP63 and CYP5141. Proline (P) is the dominant amino acid in P450 families CYP512, CYP5037, CYP5144, and CYP5152. Isoleucine (I) is the dominant amino acid among P450 families CYP5136, CYP5139, and CYP5150. This clearly suggests that each P450 family contains a pattern of amino acids characteristic of the P450 families in the P450 signature motifs EXXR and CXG. This study constitutes the first report on the identification of such P450 family-specific characteristic amino acid patterns at the EXXR and CXG motif.

### Positional Conservation or Predominance of Specific Amino Acids in the EXXR and CXG Motifs is Universal across the P450 Families in Biological Kingdoms

In order to assess the universality of the above phenomenon, we investigated member P450s of 54 P450 families from the biological kingdoms, such as animals, plants and bacteria (Table 4 and Figures S1 and S2). Sequence logos for each of the 54 P450 families constructed for EXXR (Fig. S1) and CXG (Fig. S2) motifs and analysis of the percentage conservation of the amino acid at each position in the motifs (Table 4) showed conservation or predominance of specific amino acids that display characteristics of a P450 family. Among the characteristic amino acids observed for P450 families, CYP8 showed tryptophan (W) and CYP152 showed aspartic acid (D) instead of conserved phenylalanine (F) in the CXG motif (Table 4 and Fig. S2). Although webLogos enable one to identify the amino acids that are dominant and the pattern of amino acids at the EXXR and CXG motif characteristic of the P450 family easily by looking at them (Figures S1 and S2), it is difficult to assess the data in quantitative manner. Considering this drawback, a detailed analysis of the percentage conservation of the amino acid at each position in the motifs is presented in Table 4.

**Table 4:** Comparative quantitative analysis of amino acid patterns at EXXR and CXG (FXXGXRXCXG) motifs in 54 P450 families.

P450family	Number ofmemberP450s	E-X-X-R	F-X-X-G-X-R-X-C-X-G
CYP1	288	E-T(36)/I(24)-F(66)-R	F(99.6)-G(72)-L(38)/M(38)-G(98)-K(89)-R-R(75)-C-I(89)-G
CYP2	1244	E-I(54)/V(40)-Q(93)-R	F(99)-S(93)-A(47)/I(19)/L(14)-G(99.7)-K(68)/R(24)-R(98)-I(29)/V(24)/A(18)-C-L(52)/V(16)-G(99.7)
CYP3	239	E-T(74)-L(92)-R	F(99.6)-G(99.6)-A(21)/T(23)/N(19)-G-P-R(98)-N(98)-C-I(91)-G(97)
CYP4	1034	E-S(48)/T(27)/A(20)-L(86)-R	F-S(98)-A(89)-G(99.9)-P(77)-R(98)-N(90)-C-I(92)-G(98)
CYP5	52	E-T-L-R	F-G-A-G-P-R-S-C-L(96)-G
CYP6	893	E-T(78)-L(87)-R	F(99.9)-G(98)-E(40)/D(34)-G(99.8)-P(94)-R(91)-N(50)/I(19)-C-I(89)-G(91)
CYP7	88	E-A(42)/S(41)-L(79)-R	F-G-S(60)-G-A(45)/T(24)-T(51)/S(40)-K(52)-C-P-G
CYP8	91	E-T(60)-L-R	W(96)-G-A(59)-G(97)-H(37)/V(37)-S(46)/N(45)-I(32)/H(19)/Q(16)-C-P(55)-G
CYP9	305	E-T(30)/S(25)/A(18)-L(93)-R	F-G(99)-V(30)/L(23)/I(22)-G-P(90)-R(99)-N(61)-C-I(97)-G(93)
CYP11	156	E-T(95)-L(99)-R	F-G-F(75)-G-V(49)/M(22)/P(15)-R(99)-Q-C-L(81)-G
CYP12	119	E-S(44)/G(34)-L(51)/Q(26)-R	F-G-F(96)-G-P(85)-R-M(54)/T(20)-C-I(47)/V(40)-G(98)
CYP17	99	E-V(87)-L(91)-R	F-G-A(97)-G(99)-P(61)-R-V(50)/S(48)-C-L(39)/I(32)-G
CYP19	171	E-S(72)-L(57)-R	F(99)-G(99)-S(39)/F(35)-G-P-R(99)-S(46)/A(36)-C-V(66)-G
CYP26	125	E-V(56)/T(43)-L(74)-R	F-G-G(98)-V(42)/L(36)-R-S(59)/T(28)-C-L(46)
CYP27	113	E-T(69)-L-R	F-G-Y(52)/F(33)-G-V(56)/K(28)-R-S(66)/A(29)-C-I(39)/L(34)-G
CYP53	92	E-T(52)/A(26)-L(67)/M(30)-R	F-S-H(25)/F(17)/T(17)-G-P(98)-R-A(73)-C-V(86)-G
CYP58	97	E-G(50)/S(29)/A(18)-L(59)/F(36)-R	F-S(64)-K(44)/R(39)-G-S(83)-R-Q(58)-C-I(62)-G
CYP65	203	E-A(55)/S(20)-L(65)-R	F(99)-S(77)-I(26)/V(24)/F(13)-G-P(94)-R(99)-N(80)-C-I(62)-G(98)
CYP71	767	E-T(73)-L(73)-R	F(99)-G(99)-A(57)-G(99.7)-R(96)-R(99)-I(44)/M(34)-C-P(96)-G(93)
CYP72	207	E-V(90)-L(99)-R	F-G(80)-W(79)-G-P(94)-R(99)-I(72)-C-I(72)-G(97)
CYP73	153	E-T(95)-L(95)-R	F(99)-G-V(93)-G-R-R-S(96)-C-P-G
CYP74	155	E-A(39)/T(27)/V(15)-L(88)-R	P(92)-T(53)/S(32)-V(46)/E(13)-G(32)/D(19)/S(19)-N(88)-K-Q(97)-C-A(62)-G(77)
CYP75	248	E-T(72)-F(87)-R	F-G(99.6)-A(96)-G-R(99.6)-R-I(96)-C-A(85)-G(99.6)
CYP76	201	E-T(79)-F(39)/L(37)-R	F(98)-G(97)-A(60)-G-R(93)-R(99)-I(62)-C-P(81)-G(92)
CYP78	112	E-T(51)/V(34)-L(99)-R	F-G-S(53)/A(46)-G-R(95)-R-V(68)-C-P-G
CYP79	95	E-A(78)-F(87)-R	F(99)-S(77)-T(80)-G-R(82)-R-G(96)-C-P(39)/V(17)/I(15)-G(64)
CYP81	229	E-T(89)-L(87)-R	F(99.6)-G-M(39)/L(27)-G(99.6)-R(99)-R(99.6)-A(43)/R(20)-C-P-G(94)
CYP82	166	E-T(78)-L(84)-R	F(99)-G(92)-S(74)-G(99)-R(99)-R(99)-S(46)/I(14)-C-P(96)-G(91)
CYP86	139	E-T(59)-L(86)-R	F-N(96)-A(67)-G-P-R-L(40)/T(32)-C-L(88)-G
CYP89	131	E-G(89)-L(98)-R	F-G(98)-A(71)-G(98)-R(98)-R-I(76)-C-P(75)-G(85)
CYP90	112	E-T(99)-L-R	F(89)-G-G(99)-G-P(55)/Q(36)-R-L(95)-C-P(73)-G
CYP92	163	E-T(75)-M(50)/L(36)-R	F(99)-G(99)-S(65)-G-R(99)-R-M(54)/G(26)-C-P(96)-G(91)
CYP93	150	E-T(97)-F(61)-R	F-G(99)-S(80)-G-R-R-M(51)/G(16)-C-P-G
CYP94	162	E-S(65)-M(95)-R	F-Q(62)-A(85)-G-P(74)-R-V(48)/M(22)-C-L(84)-G
CYP97	99	E-S(61)-L(64)-R	F-G(69)-G(88)-G-P(66)-R-K(91)-C-V(70)-G
CYP102	330	E-S(52)/A(27)-R	F(98)-G(99)-N(80)-G-Q(73)-R-A(94)-C-I(99)-G
CYP105	328	E-L(90)-L(81)-R	F(98)-G(93)-F(36)/Y(31)/H(28)-G-V(40)/I(28)/R(13)-H(99.7)-Q(85)-C-L(76)-G
CYP106	92	E-V(84)-L(99)-R	F-G-K(83)-G-P(85)-H-F-C-L(99)-G
CYP107	217	E-L(55)/M(15)-L(74)-R	F(92)-G(99)-H(68)-G-I(50)/V(18)-H-Y(40)/H(30)-C-L(72)-G(99)
CYP108	118	E-M(52)-I(74)-R	F-G-Y(72)-V(50)/A(26)-H-F(49)/M(19)-C-L(74)-G(99)
CYP110	113	E-T(83)-L-R	F-G-G(97)-G(88)- S(36)/N(27)/A(22)-R(99)-R(76)-C-I(62)-G(99)
CYP116	82	E-C-L-R	F-G-Y-G-S(80)-H-Q-C-M(91)-G
CYP136	137	E-S(44)/A(31)-L(86)-R	F-G-G(96)-G-A(60)/V(37)-H-K(97)-C-I(85)-G
CYP152	90	E-V(99)-R-R	D(91)-P(35)/H(20)/Y(15)-A(24)/Y(17)/E(15)-K(39)-G(63)-H-R-C-P(75)-G
CYP153	163	E-I(67)-I(94)-R	F-G-F(74)-G-I(61)-H-R-C-M(51)/V(44)-G
CYP154	104	E-T(76)-L(96)-R	F-G-H(84)-G-V(41)/P(40)-H(97)-F(22)/V(21)/H(15)-C-L(54)/P(32)-G
CYP176	102	E-V(50)/T(24)-M(60)-R	F-G(99)-G(96)-G-P(81)-R(90)-M(71)-C-P(82)-G(99)
CYP501	106	E-T(97)-L-R	F-G(97)-G(98)-G-R(98)-H-R-C-I(99)-G
CYP584	88	E-S(54)/T(22)-L(97)-R	F-N(94)-G(99)-G-P-R-I(84)-C-L(48)/I(27)-G
CYP620	167	E-V(48)/T(18)-L(68)-R	F(73)-G-F(73)-G(99)-R(99)-R-I(57)-C-P(96)-G(99)
CYP704	103	E-T(97)-L(95)-R	F-Q(76)-A(99)-G-P-R-I(81)-C-L(93)-G
CYP707	97	E-T(57)-L(89)-R	F-G(99)-N(53)-G-V(52)-T(22)-H(99)-A(51)/S(45)-C-P(99)-G(99)
CYP709	112	E-T(83)-L(96)-R	F-S(96)-L(24)/S(20)/F(16)-G(99)-P(92)-R-S(43)/V(27)-C-I(64)-G(98)
CYP716	101	E-V(49)/T(25)-M(60)-R	F-G(99)-G(96)-G-P(81)-R(90)-M(71)-C-P(83)-G(99)

The percentage predominance of amino acids at particular positions is calculated considering the total number of amino acids as 100%. Amino acids or patterns of amino acids contributing more than 50% at the specific position are shown in the table. Amino acids conserved (100%) at the specific position(s) are represented by their symbol. The numerical values in the table are percentage values.

**Figure 5:**
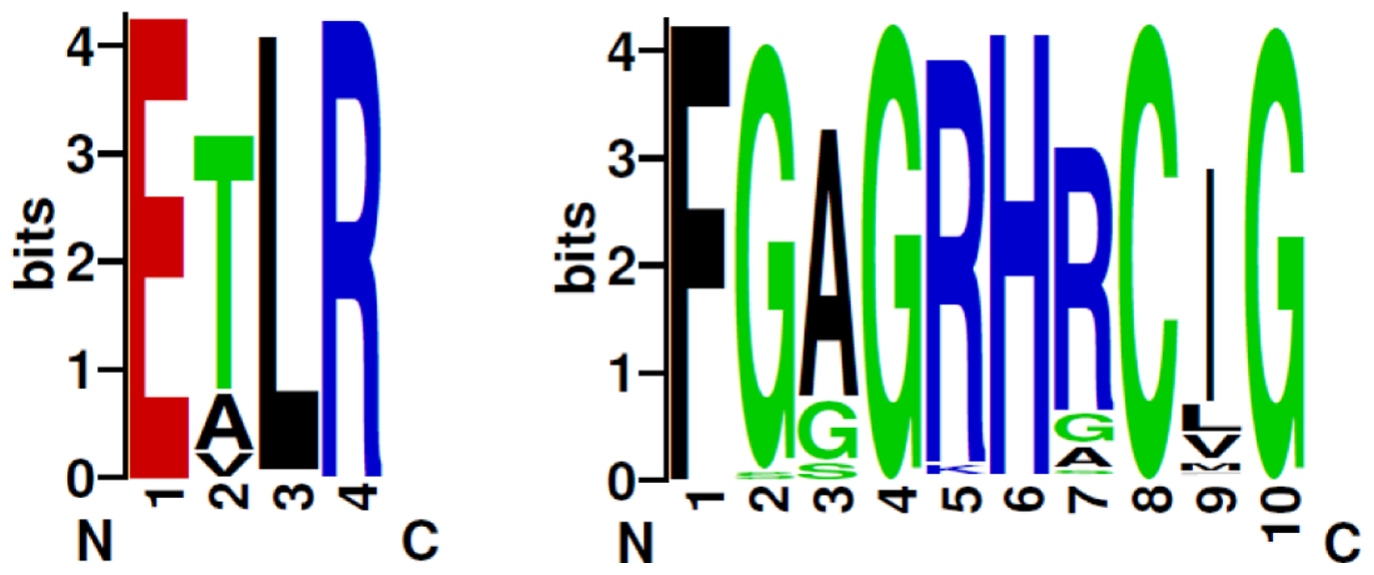
Analysis of amino acid patterns at the EXXR and CXG motif in CYP51 P450 family. In total 407 CYP51 P450 sequences representing all biological kingdoms were analyzed for EXXR and CXG signature sequences. Member P450 sequences were retrieved from the CYPED website (26) and used for analysis, as described in the “Materials and methods” section.

Detailed analysis on the dominant amino acid at each position for each family is not included in this section, considering one can easily form an impression by looking at Table 4 and Figures S1 and S2. However, we have given a detailed description of the most interesting P450 family CYP51, because this family is the only P450 family described as ubiquitous owing to its wide distribution across biological kingdoms, with one copy in most species and two copies in ascomycete and in some plants [4], [8], [58]. With its high degree of protein conservation, analysis of the EXXR and CXG motif in this P450 family was considered ideal to assess the universality of the amino acid patterns characteristic of the P450 family at these motifs. Hence, we analyzed the EXXR and CXG motif in 407 P450 sequences representing the breadth of species represented in various biological kingdoms (Fig. 5). Analysis of the CYP51 family, including the CYP51A, CYP51B, CYP51G and CYP51H subfamilies, revealed significant conservation of the EXXR and CXG motif across all CYP51 sequences independent of their biological kingdom (Fig. 5). Threonine (T) and leucine (L) are dominant and conserved amino acids at the second and third positions in the EXXR motif. Certain amino acids are dominant particularly at the “X” designated position in the consensus FXXGXRXCXG sequence (Fig. 5). Glycine (G), alanine (A) and arginine (R) are dominant amino acids at the second, third and fifth positions in this motif. Interestingly, instead of arginine (R), histidine is conserved as sixth amino acid in this motif (Fig. 5). Furthermore, the same arginine (R) amino acid is the dominant amino acid at the seventh position. Isoleucine is dominant at the ninth amino acid between conserved cysteine and glycine amino acids (Fig. 5). Considering the conservation of specific amino acid patterns at both the EXXR and CXG motifs in members of the CYP51 family, the sequence logo depicted in Fig. 5 can be considered as a characteristic amino acid pattern of the CYP51 P450 family.

### Significance of P450 Family Characteristic Amino Acid Patterns in Evolution of P450 Families

Study on the evolution of P450s and P450 families in biological kingdoms dates back to three manuscripts published in 1987 [59], [60] and 1989 [61]. The authors suggested divergent evolution of P450s from a common ancestor gene that is more than 2 billion years old [61]. The divergence of P450s occurred before eukaryote-prokaryote divergence [59]. The divergence of distinct P450 gene families from a common ancestor was estimated [62] and it was proposed arbitrarily that any P450 sharing more than 40% homology belongs to the same P450 gene family and one sharing more than 55% homology belongs to the same subfamily [44]–[46]. A large amount of data is now available on the evolution of P450s in the context of the P450 family’s distribution or duplication of P450s in different organisms. However, apart from the above homology criteria, to date no evidence has been presented or reported in the literature that distinguishes members of P450 families. It is noteworthy that P450s show functional redundancy and hence the functional properties cannot be considered as characteristic of a P450 family.

For the first time our study reports the identification of amino acid patterns in the P450 signature motifs EXXR and CXG that are characteristic of a P450 family. Analysis of 67 P450 families from biological kingdoms such as plants, animals, bacteria and fungi showed conservation of a set of amino acid patterns characteristic of a particular P450 family in EXXR and CXG motifs. This suggests that during the divergence of P450 families from a common ancestor these amino acids are conserved and retained in each P450 family as a signature of that family.

## Conclusions

In this study we performed systematic comparative analysis of the signature motifs EXXR and CXG in P450s from the large and diverse biological kingdom of fungi. The amino acids threonine and leucine are preferred at the second and third positions in the EXXR motif and proline and glycine are preferred at the second and third positions in the CXG motif in fungal P450s. This study also reports P450 family characteristics of amino acid patterns in the EXXR and CXG motif. This study is the first of its kind analyzing family-level P450 signature motifs and identifying amino acid patterns in these motifs as a signature of a P450 family. Our study constitutes the first report presenting evidence that distinguishes members of P450 families, an important aspect in P450 family divergence from a common ancestor. Results from this study open new avenues for experiments analyzing the role of these amino acid patterns in determining P450 structure, activity and/or substrate specificity that characterizes the P450 family.

## Supporting Information

Figure S1 **Analysis of amino acid patterns at EXXR motif in 54 P450 families from plants, animals, bacteria and fungi.** A sequence logo for the EXXR motif using the amino acids from 54 P450 families was constructed, as described in the “Materials and methods” section. The number of P450s used for the construction of the sequence logo is shown in the parenthesis right next to the name of the P450 family. Member P450 sequences were retrieved from CYPED (26) and used for analysis as described in the “Materials and methods” section.
(PDF)

Figure S2 **Analysis of amino acid patterns at CXG motif in 54 P450 families from plants, animals, bacteria and fungi.** A sequence logo for the CXG motif (FXXGXRXCXG) using the amino acids from 54 P450 families was constructed, as described in the “Materials and methods” section. The number of P450s used for the construction of the sequence logo is shown in the parenthesis right next to the name of the P450 family. Member P450 sequences were retrieved from CYPED website (26) and used for analysis as described in the “Materials and methods” section.
(PDF)

Table S1 **List of fungal species used in this study.** Fungal P450 sequences were obtained from published data and publicly available databases listed in the table.
(DOCX)

Table S2 **Genome data mining and selection of P450s in 12 basidiomycete species.** Proteins from each of the basidiomycete species were subjected to NCBI Batch CDD software, as described in the materials and methods sections. The proteins grouped under the P450 superfamily were selected and presented in the table. Basidiomycete species *Agaricus bisporus* and *Serpula lacrymans* P450omes were also analyzed and presented in the table.
(XLSX)

Table S3 **Comparative analysis of amino acid patterns at EXXR and CXG motifs in 4304 fungal P450s.** The amino acid patterns, number of P450s representing the pattern and percentage of the pattern are shown in the table. Percentage of pattern is calculated considering the number of P450s as 100%.
(DOCX)

Table S4 **Fungal phyla level comparative analysis of amino acid patterns at EXXR and CXG motifs in fungal P450s.** The number of P450s that showed the amino acid pattern is presented in the table.
(DOCX)
