# Supplementary figures and images for: Comparative Analysis of P450 Signature Motifs EXXR and CXG in the Large and Diverse Kingdom of Fungi: Identification of Evolutionarily Conserved Amino Acid Patterns Characteristic of P450 Family
Source: PLoS One. 2014 Apr 17;9(4):e95616. doi: 10.1371/journal.pone.0095616 (PMC3990721; doi:10.1371/journal.pone.0095616)

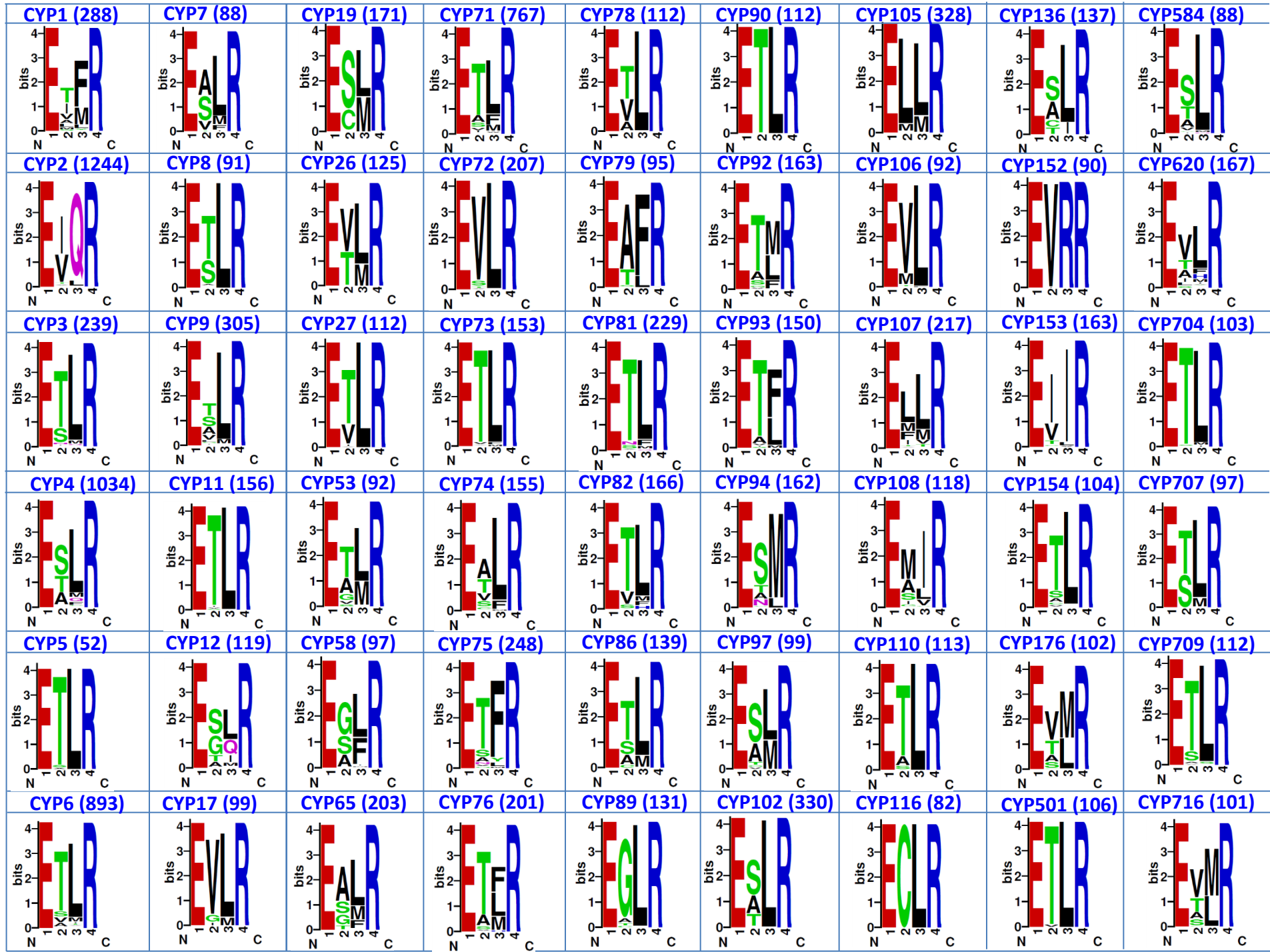

Supplement: Figure S1 — Analysis of amino acid patterns at EXXR motif in 54 P450 families from plants, animals, bacteria and fungi. A sequence logo for the EXXR motif using the amino acids from 54 P450 families was constructed, as described in the “Materials and methods” section. The number of P450s used for the construction of the sequence logo is shown in the parenthesis right next to the name of the P450 family. Member P450 sequences were retrieved from CYPED (26) and used for analysis as described in the “Materials and methods” section. (PDF) [file pone.0095616.s001.pdf]

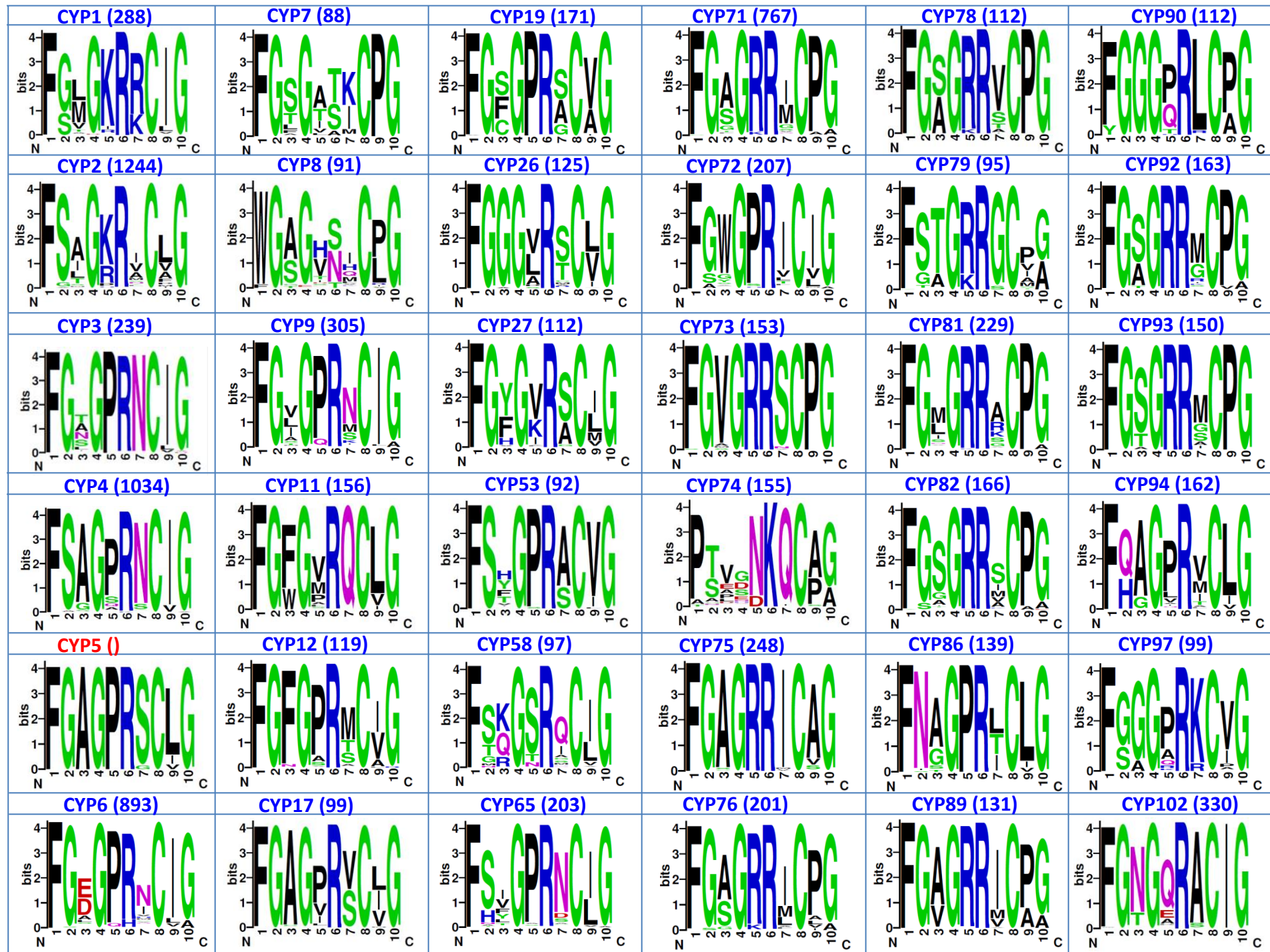

Figure S2 continued

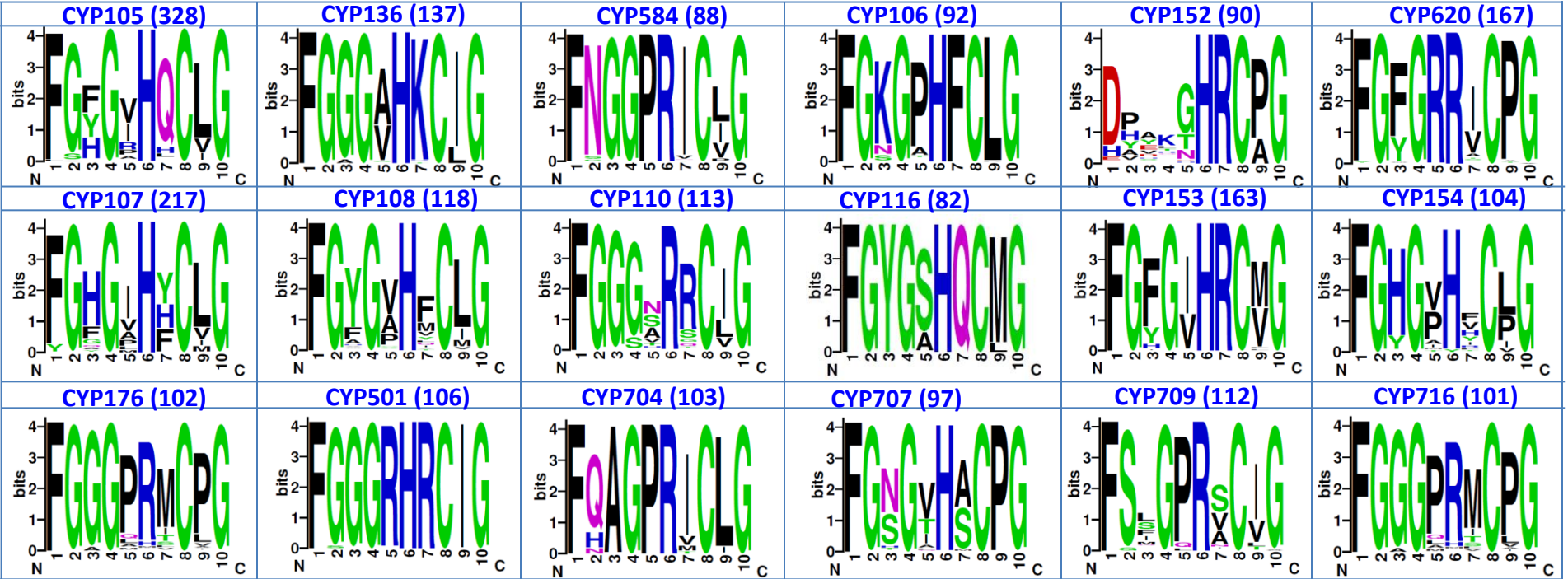

Supplement: Figure S2 — Analysis of amino acid patterns at CXG motif in 54 P450 families from plants, animals, bacteria and fungi. A sequence logo for the CXG motif (FXXGXRXCXG) using the amino acids from 54 P450 families was constructed, as described in the “Materials and methods” section. The number of P450s used for the construction of the sequence logo is shown in the parenthesis right next to the name of the P450 family. Member P450 sequences were retrieved from CYPED website (26) and used for analysis as described in the “Materials and methods” section. (PDF) [file pone.0095616.s002.pdf]
