# Supplementary material for: Comparative Analysis of P450 Signature Motifs EXXR and CXG in the Large and Diverse Kingdom of Fungi: Identification of Evolutionarily Conserved Amino Acid Patterns Characteristic of P450 Family
Source: PLoS One. 2014 Apr 17;9(4):e95616. doi: 10.1371/journal.pone.0095616 (PMC3990721; doi:10.1371/journal.pone.0095616)
Supplement: Table S1 — List of fungal species used in this study. Fungal P450 sequences were obtained from published data and publicly available databases listed in the table. (DOCX) [file pone.0095616.s003.docx]

| **Ascomycota (42 species)** | Reference | Webpage |
| --- | --- | --- |
| **Saccharomycotina (22 species)**  *Saccharomyces cerevisiae*  *Saccharomyces paradoxus*  *Saccharomyces mikatae*  *Saccharomyces kudriavzevii*  *Saccharomyces castellii*  *Saccharomyces kluyveri*  *Saccharomyces bayanus*  *Candida albicans*  *Candida tropicalis*  *Candida glabrata*  *Candida guillermondii*  *Candida parpsilosis*  *Candida dublinensis*  *Candida lusitaniae*  *Klyveromyces lactis*  *Kluyveromyces waltii*  *Kluyveromyces polysporus*  *Pichia stipitis*  *Lodderomyces elongisporus*  *Ashbya gossypii*  *Debaryomyces hansenii*  *Yarrowia lipolytica*  **Taphinomycotina (4 species)**  *Schizosaccharomyces pombe*  *Schizosaccharomyces japonicus*  *Schizosachharomyces octosporus*  *Pneumocystis carinii*  **Pezizomycotina (16 species)**  *Mycosphaerella fijiensis*  *Uncinocarpus reesii*  *Histoplasma capsulatum*  *Coccidioides immitis*  *Aspergillus clavatus*  *Aspergillus niger*  *Aspergillus flavus*  *Aspergillus oryzae*  *Aspergillus terreus*  *Aspergillus fumigatus*  *Neurospora crassa*  *Neurospora discrete*  *Fusarium graminearum*  (=*Gibberella zeae*)  *Fusarium oxysporum*  *Thielavia terrestris*  *Myceliophthora thermophila* | 3  33 | <http://drnelson.uthsc.edu/CytochromeP450.html> |
| **Basidiomycota (26 species)** |  |  |
| *Phanerochaete chrysosporium* | 34, 56, 63 | - |
| *Postia placenta* | 36, 64 | - |
| *Auricularia delicate* | 39 | <http://genome.jgi.doe.gov/Aurde1/Aurde1.home.html> |
| *Bjerkandera adusta* | 35, 65 | <http://genome.jgi-psf.org/Bjead1_1/Bjead1_1.home.html> |
| *Coniophora puteana* | 39 | <http://genome.jgi.doe.gov/Conpu1/Conpu1.home.html> |
| *Dacryopinax* sp. | 39 | <http://genome.jgi.doe.gov/Dacsp1/Dacsp1.home.html> |
| *Dichimotus squalene* | 39 | <http://genome.jgi.doe.gov/Dicsq1/Dicsq1.home.html> |
| *Fomitiporia mediterranea* | 39 | <http://genome.jgi.doe.gov/Fomme1/Fomme1.home.html> |
| *Fomitopsis pinicola* | 39 | <http://genome.jgi.doe.gov/Fompi3/Fompi3.home.html> |
| *Ganoderma* sp. | 45, 65 | <http://genome.jgi-psf.org/Gansp1/Gansp1.home.html> |
| *Gloeophyllum trabeum* | 39 | <http://genome.jgi.doe.gov/Glotr1_1/Glotr1_1.home.html> |
| *Phlebia brevispora* | 35, 65 | <http://genome.jgi-psf.org/Phlbr1/Phlbr1.home.html> |
| *Agaricus bisporus* | 24, 66 | <http://genome.jgi-psf.org/Agabi_varbisH97_2/Agabi_varbisH97_2.home.html>  and  <http://p450.riceblast.snu.ac.kr/> |
| *Punctularia strigosozonata* | 39 | <http://genome.jgi.doe.gov/Punst1/Punst1.home.html> |
| *Serpula lacrymans* | 24, 67 | <http://genome.jgi.doe.gov/SerlaS7_9_2/SerlaS7_9_2.home.html>  and  <http://p450.riceblast.snu.ac.kr/> |
| *Stereum hirsutum* | 39 | <http://genome.jgi.doe.gov/Stehi1/Stehi1.home.html> |
| *Trametes versicolor* | 39 | <http://genome.jgi.doe.gov/Trave1/Trave1.home.html> |
| *Tremella mesenterica Fries* | 39 | <http://genome.jgi-psf.org/Treme1/Treme1.home.html> |
| *Wolfiporia cocos* | 39 | <http://genome.jgi.doe.gov/Wolco1/Wolco1.home.html> |
| *Ustilago maydis* | 3 | <http://drnelson.uthsc.edu/CytochromeP450.html> |
| *Cryptococcus gattii* | 3 | <http://drnelson.uthsc.edu/CytochromeP450.html> |
| *Malassezia globosa* | 3 | <http://drnelson.uthsc.edu/CytochromeP450.html> |
| *Puccinia graminis* | 3 | <http://drnelson.uthsc.edu/CytochromeP450.html> |
| *Sporobolomyces roseus* | 3 | <http://drnelson.uthsc.edu/CytochromeP450.html> |
| *Phanerochaete carnosa* | 37 | <http://genome.jgi.doe.gov/Phaca1/Phaca1.home.html> |
| *Ganodermal lucidum* | 38 | - |
| **Zygomycota (2 species)** |  |  |
| *Rhizopus oryzae* | 3 | <http://drnelson.uthsc.edu/CytochromeP450.html> |
| *Phycomyces blakesleeanus* | 3 | <http://drnelson.uthsc.edu/CytochromeP450.html> |
| **Chytridiomycota (1 species)** |  |  |
| *Batrachochytrium dendrobatidis* | 3 | <http://drnelson.uthsc.edu/CytochromeP450.html> |

**References**

63. Martinez D, Larrondo LF, Putnam N, Gelpke MD, Huang K, et al. (2004) Genome sequence of the lignocellulose degrading fungus *Phanerochaete chrysosporium* strain RP78. Nat Biotechnol 22: 695–700.

64. Martinez D, Challacombe J, Morgenstern I, Hibbett D, Schmoll M, et al. (2009) Genome, transcriptome, and secretome analysis of the fungus *Postia placenta* supports unique mechanisms of lignocellulose conversion. Proc Natl Acad Sci USA 106: 1954–1959.

65. Binder M, Justo A, Riley R, Salamov A, Lopez-Giraldez F, et al. (2013) Phylogenetic and phylogenomic overview of the Polyporales, Mycologia 105: 1350-73.

66. Morin E, Kohler A, Baker AR, Foulongne-Oriol M, Lombard V, et al. (2012) Genome sequence of the button mushroom *Agaricus bisporus* reveals mechanisms governing adaptation to a humicrich ecological niche. Proc Natl Acad Sci USA 109: 17501-17506.

67. Eastwood DC, Floudas D, Binder M, Majcherczyk A, Schneider P, et al. (2011) The plant cell walldecomposing machinery underlies the functional diversity of forest fungi. Science 333: 762–765.
