# Supplementary material for: Comparative Analysis of P450 Signature Motifs EXXR and CXG in the Large and Diverse Kingdom of Fungi: Identification of Evolutionarily Conserved Amino Acid Patterns Characteristic of P450 Family
Source: PLoS One. 2014 Apr 17;9(4):e95616. doi: 10.1371/journal.pone.0095616 (PMC3990721; doi:10.1371/journal.pone.0095616)
Supplement: Table S3 — Comparative analysis of amino acid patterns at EXXR and CXG motifs in 4304 fungal P450s. The amino acid patterns, number of P450s representing the pattern and percentage of the pattern are shown in the table. Percentage of pattern is calculated considering the number of P450s as 100%. (DOCX) [file pone.0095616.s005.docx]

| **EXXR** | | | **CXG** | | |
| --- | --- | --- | --- | --- | --- |
| Combination | Number of P450s | percent | Combination | Number of P450s | percent |
| ETLR | 1279 | 29.72 | CPG | 1621 | 37.66 |
| EVLR | 435 | 10.11 | CIG | 1217 | 28.28 |
| ESLR | 312 | 7.25 | CLG | 708 | 16.45 |
| EALR | 305 | 7.09 | CVG | 315 | 7.32 |
| EGLR | 190 | 4.41 | CAG | 130 | 3.02 |
| ETMR | 153 | 3.55 | CSG | 55 | 1.28 |
| ESQR | 133 | 3.09 | CPA | 54 | 1.25 |
| EVYR | 92 | 2.14 | CQG | 32 | 0.74 |
| EILR | 89 | 2.07 | CTG | 31 | 0.72 |
| ECLR | 85 | 1.97 | CMG | 27 | 0.63 |
| ELLR | 83 | 1.93 | CGG | 21 | 0.49 |
| ESMR | 82 | 1.91 | CIA | 20 | 0.46 |
| EAMR | 80 | 1.86 | CKG | 11 | 0.26 |
| EVFR | 67 | 1.56 | CLA | 11 | 0.26 |
| EVMR | 56 | 1.30 | CVA | 10 | 0.23 |
| ETQR | 52 | 1.21 | CFG | 9 | 0.21 |
| ETYR | 45 | 1.05 | CEG | 6 | 0.14 |
| EVHR | 40 | 0.93 | CWG | 4 | 0.09 |
| ETIR | 35 | 0.81 | CYG | 4 | 0.09 |
| ETFR | 32 | 0.74 | CHG | 3 | 0.07 |
| EVIR | 25 | 0.58 | CRG | 3 | 0.07 |
| ELYR | 24 | 0.56 | CPK | 2 | 0.05 |
| EAFR | 21 | 0.49 | CAA | 1 | 0.02 |
| ETVR | 21 | 0.49 | CCG | 1 | 0.02 |
| EGIR | 19 | 0.44 | CID | 1 | 0.02 |
| ELFR | 19 | 0.44 | CMQ | 1 | 0.02 |
| ESSR | 19 | 0.44 | CNG | 1 | 0.02 |
| ECMR | 17 | 0.39 | CPS | 1 | 0.02 |
| ETHR | 17 | 0.39 | CSE | 1 | 0.02 |
| EAQR | 16 | 0.37 | CSP | 1 | 0.02 |
| ECQR | 16 | 0.37 | CTA | 1 | 0.02 |
| ENLR | 16 | 0.37 | CTD | 1 | 0.02 |
| ESFR | 16 | 0.37 |  | 4304 |  |
| ESIR | 16 | 0.37 |  |  |  |
| EGMR | 15 | 0.35 |  |  |  |
| EVQR | 15 | 0.35 |  |  |  |
| EAAR | 14 | 0.33 |  |  |  |
| EMLR | 13 | 0.30 |  |  |  |
| ESAR | 11 | 0.26 |  |  |  |
| EVVR | 11 | 0.26 |  |  |  |
| EAYR | 10 | 0.23 |  |  |  |
| EAIR | 9 | 0.21 |  |  |  |
| EAVR | 9 | 0.21 |  |  |  |
| ECIR | 9 | 0.21 |  |  |  |
| EIYR | 9 | 0.21 |  |  |  |
| ELCR | 9 | 0.21 |  |  |  |
| EVER | 9 | 0.21 |  |  |  |
| ESHR | 8 | 0.19 |  |  |  |
| EAHR | 7 | 0.16 |  |  |  |
| EIER | 7 | 0.16 |  |  |  |
| EIFR | 7 | 0.16 |  |  |  |
| EIHR | 7 | 0.16 |  |  |  |
| EIMR | 7 | 0.16 |  |  |  |
| ELHR | 7 | 0.16 |  |  |  |
| ELIR | 7 | 0.16 |  |  |  |
| ESER | 7 | 0.16 |  |  |  |
| EAGR | 6 | 0.14 |  |  |  |
| ECFR | 6 | 0.14 |  |  |  |
| EGFR | 6 | 0.14 |  |  |  |
| ESTR | 6 | 0.14 |  |  |  |
| ESVR | 6 | 0.14 |  |  |  |
| EASR | 5 | 0.12 |  |  |  |
| ECHR | 5 | 0.12 |  |  |  |
| ECVR | 5 | 0.12 |  |  |  |
| EGVR | 5 | 0.12 |  |  |  |
| ELSR | 5 | 0.12 |  |  |  |
| ENFR | 5 | 0.12 |  |  |  |
| ETGR | 5 | 0.12 |  |  |  |
| ETSR | 5 | 0.12 |  |  |  |
| EGQR | 4 | 0.09 |  |  |  |
| EIIR | 4 | 0.09 |  |  |  |
| ELTR | 4 | 0.09 |  |  |  |
| EMYR | 4 | 0.09 |  |  |  |
| ENIR | 4 | 0.09 |  |  |  |
| ESGR | 4 | 0.09 |  |  |  |
| ETAR | 4 | 0.09 |  |  |  |
| EVTR | 4 | 0.09 |  |  |  |
| EATR | 3 | 0.07 |  |  |  |
| ECAR | 3 | 0.07 |  |  |  |
| ECSR | 3 | 0.07 |  |  |  |
| ENGR | 3 | 0.07 |  |  |  |
| ENMR | 3 | 0.07 |  |  |  |
| ENSR | 3 | 0.07 |  |  |  |
| ESYR | 3 | 0.07 |  |  |  |
| ETCR | 3 | 0.07 |  |  |  |
| ETNR | 3 | 0.07 |  |  |  |
| EVAR | 3 | 0.07 |  |  |  |
| EAWR | 2 | 0.05 |  |  |  |
| ECRR | 2 | 0.05 |  |  |  |
| ECTR | 2 | 0.05 |  |  |  |
| EEGR | 2 | 0.05 |  |  |  |
| EELR | 2 | 0.05 |  |  |  |
| EFER | 2 | 0.05 |  |  |  |
| EFHR | 2 | 0.05 |  |  |  |
| EIQR | 2 | 0.05 |  |  |  |
| EIVR | 2 | 0.05 |  |  |  |
| ELNR | 2 | 0.05 |  |  |  |
| EMFR | 2 | 0.05 |  |  |  |
| ESNR | 2 | 0.05 |  |  |  |
| ETTR | 2 | 0.05 |  |  |  |
| EVCR | 2 | 0.05 |  |  |  |
| EVRR | 2 | 0.05 |  |  |  |
| EVSR | 2 | 0.05 |  |  |  |
| EVWR | 2 | 0.05 |  |  |  |
| EACR | 1 | 0.02 |  |  |  |
| EANR | 1 | 0.02 |  |  |  |
| EAPR | 1 | 0.02 |  |  |  |
| EFQR | 1 | 0.02 |  |  |  |
| EFVR | 1 | 0.02 |  |  |  |
| EGAR | 1 | 0.02 |  |  |  |
| EGGR | 1 | 0.02 |  |  |  |
| EGHR | 1 | 0.02 |  |  |  |
| EGNR | 1 | 0.02 |  |  |  |
| EGTR | 1 | 0.02 |  |  |  |
| ELMR | 1 | 0.02 |  |  |  |
| EMMR | 1 | 0.02 |  |  |  |
| EMTR | 1 | 0.02 |  |  |  |
| ENTR | 1 | 0.02 |  |  |  |
| EPMR | 1 | 0.02 |  |  |  |
| EQLR | 1 | 0.02 |  |  |  |
| ESDR | 1 | 0.02 |  |  |  |
| ESPR | 1 | 0.02 |  |  |  |
| ESWR | 1 | 0.02 |  |  |  |
| ETRR | 1 | 0.02 |  |  |  |
| ETWR | 1 | 0.02 |  |  |  |
| EVGR | 1 | 0.02 |  |  |  |
| EVLS | 1 | 0.02 |  |  |  |
| EWKR | 1 | 0.02 |  |  |  |
|  | 4304 |  |  |  |  |
