# Supplementary material for: Comparative Analysis of P450 Signature Motifs EXXR and CXG in the Large and Diverse Kingdom of Fungi: Identification of Evolutionarily Conserved Amino Acid Patterns Characteristic of P450 Family
Source: PLoS One. 2014 Apr 17;9(4):e95616. doi: 10.1371/journal.pone.0095616 (PMC3990721; doi:10.1371/journal.pone.0095616)
Supplement: Table S4 — Fungal phyla level comparative analysis of amino acid patterns at EXXR and CXG motifs in fungal P450s. The number of P450s that showed the amino acid pattern is presented in the table. (DOCX) [file pone.0095616.s006.docx]

| Ascomycotina | | | | Basidiomycota |  | Zygomycota |  | Chytridiomycota |  |
| --- | --- | --- | --- | --- | --- | --- | --- | --- | --- |
| Pezizomycotina |  | Saccharomycotina & Taphinomycotina |  |  |  |  |  |  |  |
| Combination | Count | Combination | Count | Combination | Count | Combination | Count | Combination | Count |
| **EXXR** | | | | | | | | | |
| ETLR | 262 | ETLR | 84 | ETLR | 897 | ETLR | 34 | EAQR | 2 |
| ESLR | 136 | EALR | 14 | EVLR | 316 | EVLR | 13 | ETLR | 2 |
| EALR | 115 | ECLR | 14 | EALR | 174 | ENLR | 12 | ESLR | 1 |
| EVLR | 100 | ESLR | 12 | ESLR | 158 | ETMR | 6 | ESQR | 1 |
| EGLR | 81 | EILR | 6 | EGLR | 104 | ESLR | 5 | ETGR | 1 |
| EAMR | 51 | EVLR | 6 | ETMR | 102 | ENIR | 4 |  | 7 |
| ESQR | 47 | EAVR | 3 | EVYR | 89 | ECLR | 3 |  |  |
| ETMR | 44 | EGLR | 3 | ESQR | 85 | EIMR | 3 |  |  |
| ESMR | 33 | ENSR | 3 | EILR | 67 | EALR | 2 |  |  |
| ETQR | 33 | ETVR | 3 | ELLR | 60 | EGLR | 2 |  |  |
| ELLR | 23 | ECIR | 2 | EVFR | 56 | EILR | 2 |  |  |
| ECLR | 19 | ENGR | 2 | EVMR | 52 | ETFR | 2 |  |  |
| EGIR | 16 | EVVR | 2 | ECLR | 49 | ETIR | 2 |  |  |
| EAFR | 14 | EACR | 1 | ESMR | 47 | EVQR | 2 |  |  |
| EILR | 14 | EMLR | 1 | ETYR | 42 | ECIR | 1 |  |  |
| EAQR | 11 | ENLR | 1 | EVHR | 40 | ENGR | 1 |  |  |
| EVFR | 10 | ENTR | 1 | EAMR | 29 | ENMR | 1 |  |  |
| ELCR | 9 | ESMR | 1 | ETIR | 29 | ESAR | 1 |  |  |
| ESIR | 9 | ETIR | 1 | ELYR | 24 | ESMR | 1 |  |  |
| ETFR | 9 | ETMR | 1 | ETFR | 21 | ESSR | 1 |  |  |
| ETVR | 9 | ETSR | 1 | ETQR | 19 | ESVR | 1 |  |  |
| ESAR | 7 |  | 162 | EVIR | 19 | EVFR | 1 |  |  |
| EAVR | 6 |  |  | ECMR | 17 | EVIR | 1 |  |  |
| EAIR | 5 |  |  | ELFR | 16 | EVMR | 1 |  |  |
| EGMR | 5 |  |  | ESSR | 15 |  | 102 |  |  |
| ESFR | 5 |  |  | EAAR | 14 |  |  |  |  |
| ETHR | 5 |  |  | ECQR | 12 |  |  |  |  |
| EVIR | 5 |  |  | ETHR | 12 |  |  |  |  |
| EAGR | 4 |  |  | ESFR | 11 |  |  |  |  |
| EAHR | 4 |  |  | EVQR | 11 |  |  |  |  |
| ECQR | 4 |  |  | EGMR | 10 |  |  |  |  |
| EGFR | 4 |  |  | EAYR | 9 |  |  |  |  |
| EMLR | 4 |  |  | ETVR | 9 |  |  |  |  |
| ENFR | 4 |  |  | EIYR | 8 |  |  |  |  |
| ELFR | 3 |  |  | EMLR | 8 |  |  |  |  |
| ESSR | 3 |  |  | EVVR | 8 |  |  |  |  |
| ESVR | 3 |  |  | EAFR | 7 |  |  |  |  |
| ETIR | 3 |  |  | EIER | 7 |  |  |  |  |
| ETYR | 3 |  |  | EIHR | 7 |  |  |  |  |
| EVMR | 3 |  |  | ELHR | 7 |  |  |  |  |
| EVYR | 3 |  |  | ELIR | 7 |  |  |  |  |
| EAWR | 2 |  |  | ESER | 7 |  |  |  |  |
| ECIR | 2 |  |  | ESHR | 7 |  |  |  |  |
| ECVR | 2 |  |  | ESIR | 7 |  |  |  |  |
| EEGR | 2 |  |  | EVER | 7 |  |  |  |  |
| ELSR | 2 |  |  | EIFR | 6 |  |  |  |  |
| ENMR | 2 |  |  | ECFR | 5 |  |  |  |  |
| ETCR | 2 |  |  | ECHR | 5 |  |  |  |  |
| ETNR | 2 |  |  | ESTR | 5 |  |  |  |  |
| EVER | 2 |  |  | EAIR | 4 |  |  |  |  |
| EVQR | 2 |  |  | EASR | 4 |  |  |  |  |
| EANR | 1 |  |  | ECIR | 4 |  |  |  |  |
| EASR | 1 |  |  | EGQR | 4 |  |  |  |  |
| EAYR | 1 |  |  | EGVR | 4 |  |  |  |  |
| ECAR | 1 |  |  | EIIR | 4 |  |  |  |  |
| ECFR | 1 |  |  | EIMR | 4 |  |  |  |  |
| EELR | 1 |  |  | ELTR | 4 |  |  |  |  |
| EFVR | 1 |  |  | EMYR | 4 |  |  |  |  |
| EGGR | 1 |  |  | ETAR | 4 |  |  |  |  |
| EGHR | 1 |  |  | EVTR | 4 |  |  |  |  |
| EGNR | 1 |  |  | EAHR | 3 |  |  |  |  |
| EGTR | 1 |  |  | EAQR | 3 |  |  |  |  |
| EGVR | 1 |  |  | EATR | 3 |  |  |  |  |
| EIFR | 1 |  |  | ECSR | 3 |  |  |  |  |
| EIQR | 1 |  |  | ECVR | 3 |  |  |  |  |
| EIYR | 1 |  |  | EGIR | 3 |  |  |  |  |
| ELMR | 1 |  |  | ELSR | 3 |  |  |  |  |
| EPMR | 1 |  |  | ENLR | 3 |  |  |  |  |
| ESGR | 1 |  |  | ESAR | 3 |  |  |  |  |
| ESHR | 1 |  |  | ESGR | 3 |  |  |  |  |
| ESPR | 1 |  |  | ESYR | 3 |  |  |  |  |
| ESTR | 1 |  |  | ETGR | 3 |  |  |  |  |
| ESWR | 1 |  |  | ETSR | 3 |  |  |  |  |
| ETGR | 1 |  |  | EVAR | 3 |  |  |  |  |
| ETSR | 1 |  |  | EAGR | 2 |  |  |  |  |
| ETTR | 1 |  |  | ECAR | 2 |  |  |  |  |
| EVVR | 1 |  |  | ECRR | 2 |  |  |  |  |
|  | 1174 |  |  | ECTR | 2 |  |  |  |  |
|  |  |  |  | EFER | 2 |  |  |  |  |
|  |  |  |  | EFHR | 2 |  |  |  |  |
|  |  |  |  | EGFR | 2 |  |  |  |  |
|  |  |  |  | EIVR | 2 |  |  |  |  |
|  |  |  |  | ELNR | 2 |  |  |  |  |
|  |  |  |  | EMFR | 2 |  |  |  |  |
|  |  |  |  | ESNR | 2 |  |  |  |  |
|  |  |  |  | ESVR | 2 |  |  |  |  |
|  |  |  |  | EVCR | 2 |  |  |  |  |
|  |  |  |  | EVRR | 2 |  |  |  |  |
|  |  |  |  | EVSR | 2 |  |  |  |  |
|  |  |  |  | EVWR | 2 |  |  |  |  |
|  |  |  |  | EAPR | 1 |  |  |  |  |
|  |  |  |  | EELR | 1 |  |  |  |  |
|  |  |  |  | EFQR | 1 |  |  |  |  |
|  |  |  |  | EGAR | 1 |  |  |  |  |
|  |  |  |  | EIQR | 1 |  |  |  |  |
|  |  |  |  | EMMR | 1 |  |  |  |  |
|  |  |  |  | EMTR | 1 |  |  |  |  |
|  |  |  |  | ENFR | 1 |  |  |  |  |
|  |  |  |  | EQLR | 1 |  |  |  |  |
|  |  |  |  | ESDR | 1 |  |  |  |  |
|  |  |  |  | ETCR | 1 |  |  |  |  |
|  |  |  |  | ETNR | 1 |  |  |  |  |
|  |  |  |  | ETRR | 1 |  |  |  |  |
|  |  |  |  | ETTR | 1 |  |  |  |  |
|  |  |  |  | ETWR | 1 |  |  |  |  |
|  |  |  |  | EVGR | 1 |  |  |  |  |
|  |  |  |  | EVLS | 1 |  |  |  |  |
|  |  |  |  | EWKR | 1 |  |  |  |  |
|  |  |  |  |  | 2859 |  |  |  |  |
| **CXG** | | | | | | | | | |
| CIG | 370 | CLG | 122 | CPG | 1329 | CIG | 37 | CIG | 5 |
| CLG | 268 | CIG | 28 | CIG | 777 | CPG | 33 | CLG | 2 |
| CPG | 258 | CSG | 7 | CLG | 292 | CLG | 24 |  | 7 |
| CVG | 88 | CAG | 2 | CVG | 220 | CVG | 6 |  |  |
| CAG | 44 | CCG | 1 | CAG | 84 | CPA | 1 |  |  |
| CPA | 23 | CPG | 1 | CPA | 30 | CQG | 1 |  |  |
| CSG | 19 | CVG | 1 | CSG | 29 |  | 102 |  |  |
| CIA | 17 |  | 162 | CQG | 22 |  |  |  |  |
| CMG | 17 |  |  | CGG | 20 |  |  |  |  |
| CTG | 15 |  |  | CTG | 16 |  |  |  |  |
| CLA | 9 |  |  | CMG | 10 |  |  |  |  |
| CQG | 9 |  |  | CEG | 5 |  |  |  |  |
| CKG | 8 |  |  | CVA | 5 |  |  |  |  |
| CFG | 5 |  |  | CFG | 4 |  |  |  |  |
| CVA | 5 |  |  | CIA | 3 |  |  |  |  |
| CWG | 4 |  |  | CKG | 3 |  |  |  |  |
| CHG | 2 |  |  | CYG | 3 |  |  |  |  |
| CPK | 2 |  |  | CLA | 2 |  |  |  |  |
| CRG | 2 |  |  | CHG | 1 |  |  |  |  |
| CAA | 1 |  |  | CMQ | 1 |  |  |  |  |
| CEG | 1 |  |  | CPS | 1 |  |  |  |  |
| CGG | 1 |  |  | CRG | 1 |  |  |  |  |
| CID | 1 |  |  | CSE | 1 |  |  |  |  |
| CNG | 1 |  |  |  | 2859 |  |  |  |  |
| CSP | 1 |  |  |  |  |  |  |  |  |
| CTA | 1 |  |  |  |  |  |  |  |  |
| CTD | 1 |  |  |  |  |  |  |  |  |
| CYG | 1 |  |  |  |  |  |  |  |  |
|  | 1174 |  |  |  |  |  |  |  |  |
|  |  |  |  |  |  |  |  |  |  |
